# Perceptions of a Diverse Group of U.S. Women on the Ease of Vaginal Self-Sampling for Cancer Detection

**DOI:** 10.1177/26884844251379035

**Published:** 2025-09-18

**Authors:** Parker O’Connell, Roaa Rafat Mohamed, Amy Delicia Akineza, Arnaud Iradukunda, Christina Burns, Ozlem Equils

**Affiliations:** ^1^ MiOra, Los Angeles, California, USA.; ^2^ Eastern Virginia Medical School, Norfolk, Virginia, USA.; ^3^ Ahfad University for Women, School of Medicine, Omdurman, Sudan.; ^4^ University of Burundi, Faculty of Medicine, Bujumbura, Burundi.; ^5^ Department of Research and Innovation , ARNECH Research and Consulting Office, Bujumbura, Burundi.; ^6^ Kaiser Permanente Bernard J. Tyson School of Medicine, Pasadena, CA, USA.

**Keywords:** cervical cancer, education, HPV, Pap smear, preventive care, women’s health

## Abstract

**Introduction:**

The recent Food and Drug Administration approval of a cervical self-collection method for human papillomavirus detection offers a promising opportunity to improve access to cervical cancer screenings. This study evaluates patients’ perceptions of self-collection methods and identifies factors influencing their acceptance.

**Methods:**

MiOra health educators conducted a pilot, cross-sectional, convenience sampling study in Los Angeles County, California, using an Institutional Review Board-approved Qualtrics electronic survey targeting low socioeconomic women. Participants evaluated the perceived ease or difficulty of at-home self-collection methods for vaginal and nasopharyngeal swabs. Associations between sociodemographic, behavioral, and contextual factors with self-sampling preferences were analyzed using chi-square test. Statistical significance was set at 5%, and data were analyzed in R version 4.4.1.

**Results:**

A total of 213 women aged 18 years and older participated in the study, with no exclusions. The majority of participants were under 51 years old (83.6%), Hispanic/Latino (61.5%), and first-generation immigrants (54.5%) in the United States. Women with a middle school or less education were significantly more likely to report perceived difficulty with vaginal self-collection as compared with women with a graduate or professional school education (85.7% vs. 21.4%, respectively, *p* = 0.009).

**Conclusion:**

Timely cervical cancer diagnosis is crucial for improving treatment outcomes. Findings from this pilot study suggest that formal education may influence women’s comfort level with vaginal self-collection. Further research is needed to understand the role of formal education to close the gaps in timely cancer detection.

## Introduction

Cervical cancer is the fourth most common cancer in women globally,^1^ with significant disparities in morbidity and mortality due to limited human papillomavirus (HPV) vaccination access and delayed diagnosis.^2^ In the United States, cervical cancer accounts for over 13,000 new cases and 4,000 deaths annually, underscoring the critical need for early detection.^3^ The World Health Organization (WHO) recommends that screening for cervical cancer begins at 30 years of age, repeated every 5–10 years, and for those diagnosed with human immunodeficiency virus beginning at 25 years of age, repeated every 3–5 years.^4^ Similarly, the American College of Obstetricians and Gynecologists (ACOG) has adopted the U.S. Preventive Services Task Force guidelines, which recommend Pap testing every 3 years for women aged 21–29 and primary high risk (hr)HPV testing every 5 years for average-risk patients aged 25–29 years based on its Food and Drug Administration (FDA)-approved age for use and primary hrHPV testing’s demonstrated efficacy in individuals aged 25 years and older. For women 30–65 years old, ACOG recommends three options for cervical cancer screening: primary hrHPV testing every 5 years, cervical cytology alone every 3 years, or co-testing with a combination of cytology and hrHPV testing every 5 years.^5,6^ Over 90% of cervical cancer cases are caused by HPV infection, and screening via Pap smear reduces incidence and mortality by up to 80%.^7,8^ However, nearly 50% of cervical cancer cases occur in individuals with insufficient screening,^9^ driven by barriers such as cultural beliefs, ethnicity, and a history of trauma.^10,11^

Cultural norms surrounding virginity and modesty in various communities discourage screening, particularly among Muslim, Latina, and other non-White populations.^12–14^ Structural barriers, including lack of access, insurance, and systemic racism, further limit screening rates, leading to advanced-stage diagnoses and higher mortality in these groups.^15,16^ Survivors of sexual abuse often avoid gynecological exams due to fear, discomfort, and trauma triggers, creating an additional barrier to care.^17–19^

The FDA’s 2024 approval of HPV self-collection methods offers a promising alternative, enabling individuals to collect cervical samples without a speculum in a health care setting such as primary care offices, urgent care, pharmacies, and mobile clinics.^20^ HPV self-collection has comparable performance with a physician-collected sample, in the Cervical and Self-Sample In a Screening study, high-grade cervical intraepithelial neoplasia was detected with positive predictive values of 28% and 29.7%, respectively.^21^ In 2021, the WHO guideline on self-care interventions for health and well-being includes self-sampling as part of the cervical cancer screening guidelines.^22^ Despite this fact, HPV self-collection is still limited; meta-analyses identified only 17 countries that recommend its use in the screening programs, nine as the primary collection method, and eight to reach under-screened populations.^23^ The newly approved self-collection tests bring the United States closer to countries where self-collected samples are widely used, including the Netherlands, Denmark, Sweden, and Australia.^20,24,25^ In addition, screening access will be further widened by the newly launched clinical trial, called self-collection for HPV testing to improve cervical cancer prevention, to test for in-home self-collection.^20^

During the coronavirus disease 2019 (COVID-19) pandemic, self-collection became widely accepted for diagnostic purposes, demonstrating ease, comfort, and reliability.^26,27^ Vaginal self-collection has also been effective for diagnosing infections, with high patient satisfaction and willingness to recommend the method.^28,29^

This study evaluates patients’ perceptions of vaginal self-collection and identifies factors influencing its acceptance. By addressing barriers to traditional screening, self-collection could expand access and reduce cervical cancer disparities.

## Methods

This is a pilot study using data generated from a cross-sectional study conducted in Los Angeles County, California, between May and July 2023 to assess minority women’s attitudes toward female reproductive health and health literacy (Supplementary Appendix A1). The survey for the parent study included questions on the comfort level of responding women with self-collection for vaginal sample for cervical cancer screening. Low levels of knowledge of HPV infection and cervical cancer contribute to low cervical cancer screening rates, and early health education can significantly increase the cervical screening rates.^30^ A recent study showed that in women aged 21-29 there has been a decline in cytology testing for cervical cancer, with annual testing use of 29.4% in 2019, down 23% from 2013. ^31^ Although they are outside of the ACOG-recommended cervical cancer screening age range, we recruited women 18 years and older for this study to determine their attitudes and for community mobilization.^32^ We used convenience sampling through electronic surveys (developed in Qualtrics) and in-person distribution at health fairs by MiOra health educators, who were students from California State University, Los Angeles, or Long Beach Departments of Public Health or Health Literacy. No personal identifier was collected. This study was determined to be exempt from further review by the Western Institutional Review Board (1-1653595-1).

Participants’ willingness and ease or difficulty with self-collection for vaginal and nasal samples were assessed. Independent variables included sociodemographic factors (age, ethnicity, education, and immigration status), health care access (insurance, family/friend support), and comfort with male or female health care providers. Descriptive statistics summarized responses, and chi-square tests examined associations between these factors and self-collection perceptions. Statistical significance was set at *p* < 0.05, and analyses were performed using R version 4.4.1.

## Results

A total of 213 women aged 18 years and older participated in the study, with no exclusions. The majority of participants were under 51 years old (83.6%), Hispanic/Latino (61.5%), and first-generation immigrants in the United States (54.5%) (Table 1). About one-third (33%) had completed 4 years of college, 92.5% had health insurance, and a large majority reported having family or social support (94.8%) (Table 1). However, 25.8% of participants reported not attending a yearly female health appointment (Table 1).

**Table 1.** Vaginal Self Collection Acceptability

		Vaginal self collection		
Variable	*N* (%)	Easy (%)	Difficult (%)	*p* value
Age				0.103
18–50	178 (83.6%)	68.5	31.5	
51+	35 (16.4%)	54.3	45.7	
Ethnicity				0.062
American Indian or Alaska Native	2 (0.9%)	100.0	0.0	
Asian and Pacific Islander Hawaiian	38 (17.8%)	63.2	36.8	
Black or African American	17 (8.0%)	82.4	17.6	
Hispanic/Latino	131 (61.5%)	60.3	39.7	
Other	7 (3.3%)	85.7	14.3	
White	18 (8.5%)	88.9	11.1	
First-generation immigrant				0.092
No	97 (45.5%)	72.2	27.8	
Yes	116 (54.5%)	61.2	38.8	
Educational level				0.009
1–2 years college	41 (19.2%)	70.7	29.3	
4 years college	71 (33.3%)	67.6	32.4	
Graduate/Professional school	42 (19.7%)	78.6	21.4	
High school/GED	45 (21.1%)	64.4	35.6	
Middle school or less	14 (6.6%)	14.3	85.7	
Strong support from family/friends				0.135
No	11 (5.2%)	45.5	54.5	
Yes	202 (94.8%)	67.3	32.7	
Yearly female health appointment attendance, which type of provider				0.283
No	55 (25.8%)	70.9	29.1	
Yes, family doctor	99 (46.5%)	59.6	40.4	
Yes, gynecologist	54 (25.4%)	72.2	27.8	
Yes, nurse	5 (2.3%)	80.0	20.0	
Health insurance				0.154
No	16 (7.5%)	50.0	50.0	
Yes	197 (92.5%)	67.5	32.5	
Advice on sexual and/or genital organ health				0.519
Family member	18 (8.5%)	61.1	38.9	
Friend	11 (5.2%)	81.8	18.2	
Nurse	12 (5.6%)	58.3	41.7	
Obstetrician	52 (24.4%)	67.3	32.7	
Online medical educator	5 (2.3%)	80.0	20.0	
Physician	107 (50.2%)	67.3	32.7	
Social media and/or Internet posts	8 (3.8%)	37.5	62.5	
Equally as comfortable asking a male obstetrician, nurse, or physician about genital health advice, as a female one				0.002
Yes	65 (30.5%)	78.5	21.5	
No, more comfortable with female	120 (56.3%)	62.5	37.5	
No, more comfortable with male	5 (2.3%)	0.0	100.0	

GED, general educational development.

Hispanic/Latino women (39.7%, *p* = 0.062) and Asian/Pacific Islander Hawaiian women (36.8%, *p* = 0.062), women with middle school or less education (85.7%, *p* = 0.009), without strong support from family (54.5% *p* = 0.135), and without health insurance (50%, *p* = 0.154) were more likely to report that vaginal self-collection would be difficult (Table 1). Regarding health care provider comfort, about half of participants (56.3%, *p* = 0.002) did not feel as comfortable seeking genital health advice from a male obstetrician, nurse, or physician as they did from a female provider (Table 1). In contrast, 30.5% reported feeling equally comfortable with both male and female providers (Table 1). Physicians were the preferred source for sexual and genital health advice for 50.2% of participants, with the remainder seeking advice from other sources, such as family, friends, and social media. Out of 72 women who believe vaginal self-collection would be difficult, the majority (62.5%) reported using social media and/or internet posts for advice on sexual/genital organ health (Table 1).

## Discussion

The goal of this pilot study was to evaluate women’s attitudes on the perceived difficulty of self-collection of vaginal samples for cervical cancer screening and the factors that may influence their response.

Our findings indicate that lower formal educational attainment was significantly associated with greater perceived difficulty in vaginal self-collection. Groups with the highest rates of believing vaginal self-collection would be difficult were participants with middle school or less educational background. Sexual health education, often provided in school settings, is a way for children to learn about their bodies and, therefore, their health^33^ as an active form of personal health literacy.^34^ A study surveying women in Egypt shows a strong correlation between health literacy and cervical cancer screening knowledge, indicating the two are intimately connected.^35^ This suggests that sexual health education provided in school settings can influence a person’s comfortability and understanding of their body, potentially leading to increased acceptability with cervical self-collection.

The concept of self-collection of vaginal samples can be compared with self-collection of nasal or oropharyngeal samples, a popular diagnostic method during the COVID-19 pandemic.^36,37^ In our study, 96.2% of the respondents reported that it is easy to collect nasal samples (Table 2). Building on training models used for self-collection of nasopharyngeal samples during the COVID-19 pandemic could be an effective approach to educating women on self-collection of vaginal samples for cervical cancer screening. A study in October 2020 found that infographics instructing patients on nasal swabs led to a high level of compliance across various demographics, with patients rating the ease and comfort greater than 79%.^38^ The WHO recommends that the information on self-testing be concise, age-appropriate, and context-specific, with supportive materials such as hotlines, brochures, or web-based video instruction.^39^ For example, a study of 197 symptomatic children and adolescents who used self-collected nasal swabs following simple instructions demonstrated high agreement (97.8% of positive severe acute respiratory syndrome coronavirus 2 samples) with results following collection by health care workers.^40^ Such models could be adapted for cervical cancer screening to improve acceptability and increase participation rates.

**Table 2.** Nasal Self-Collection Acceptability

		Nasal self-collection		
Variable	*N* (%)	Easy (%)	Difficult (%)	*p* value
Educational level				0.215
1–2 years college	41 (19.2%)	95.1%	4.9%	
4 years college	71 (33.3%)	98.6%	1.4%	
Graduate/Professional school	42 (19.7%)	97.6%	2.4%	
High school/GED	45 (21.1%)	95.6%	4.4%	
Middle school or less	14 (6.6%)	85.7%	14.3%	

GED, general educational development.

Although our results did not show a significant correlation between age and perceived difficulty, women over 50 years of age were more likely to report challenges with vaginal sample collection. This may be due to factors such as a perceived lower risk of cervical cancer, particularly among those without a sexual partner or who are not sexually active. Providing education on the long timeframe between HPV infection and cervical cancer development could help address these misconceptions. For example, a study by Marlow et al. found that explaining this extended interval can increase the perceived importance of screening among older women.^41^

The variability in perceived difficulty with vaginal self-collection may stem from the vulnerability associated with the procedure. Sexual violence is prevalent, with more than half of women experiencing sexual assault in their lifetime.^42^ Similar to the practice of allowing patients to insert the speculum themselves during pelvic exams to enhance comfort, cervical self-collection offers a sense of autonomy and control, which is especially important for survivors of sexual assault.^43^

Additionally, in many conservative cultures, the emphasis on virginity can create barriers to adequate cervical screening. To address these concerns, health care providers should offer education on the procedure within the cultural context of virginity and purity. Incorporating cultural competency and empathy into gynecological training and clinical practice is essential to providing effective, patient-centered care that respects diverse cultural perspectives.

### Limitations

This pilot study has several limitations. Its cross-sectional design prevents the establishment of causal relationships or tracking changes in perception over time. Additionally, while the study included women from minority groups, its limited geographic scope restricts the generalizability of the findings to all women. Further research with a broader sample is needed to determine how these results apply to women from diverse cultural, educational, and geographical backgrounds. Future research should also explore patients’ experiences after performing vaginal self-collection. Although this study was not designed to compare self-collection for cervical cancer screening with COVID-19 screening, the findings still provide valuable insights. Lastly, as a U.S.-based study, the results may not be generalizable to other countries.

## Conclusion

Cervical cancer remains one of the most commonly diagnosed cancers and is traditionally screened through Pap smears, where a health care provider collects a cervical sample to detect HPV. The recent FDA approval of cervical self-collection presents an opportunity to reduce screening disparities by improving access and compliance across diverse age groups, racial and ethnic communities, education levels, and among survivors of sexual trauma. Findings from this pilot study indicate that women with lower education levels (middle school or less) were more likely to perceive vaginal self-collection as difficult. Integrating cervical self-collection with targeted educational efforts could enhance access to and participation in cervical cancer screening, ultimately improving early detection and health outcomes.

## Acknowledgment

The authors are grateful for the team at MiOra for their support and guidance on this project. The authors thank Marisa Munoz, Kalkidan Amare, Andrea Robertson, Aileen Valenzuela, Stephanie Ortiz, Kenia Yvonne Villalobos, Andrea Matal, and Ricardo Ortiz Baca for their participation in the survey data collection.

## Notes

Supplementary Appendix A1

## Abbreviations Used

COVID-19: coronavirus disease 2019
FDA: Food and Drug Administration
GED: general educational development
HPV: human papillomavirus
WHO: World Health Organization
