# Supplementary material for: Perceptions of a Diverse Group of U.S. Women on the Ease of Vaginal Self-Sampling for Cancer Detection
Source: Womens Health Rep (New Rochelle). 2025 Sep 18;6(1):912–8. doi: 10.1177/26884844251379035 (PMC12547394; doi:10.1177/26884844251379035)
Supplement: Supplementary Appendix A1 [file 26884844251379035_supplementary_appendix_a1.docx]

Understanding the Awareness and Knowledge of Ovarian & Endometrial Cancer in the LA Community

Start of Block: Welcome!

Q35 Please enter your anonymous respondent number below (provided to you by your MiOra Health Ambassador).

________________________________________________________________

End of Block: Welcome!

Start of Block: Pre-Education Questions

Q1 What is your age?

- 18-25 (1)
- 26-30 (2)
- 31-40 (3)
- 41-50 (4)
- 51-60 (5)
- 61+ (6)

| 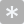 |
| --- |

Q36 What is your assigned gender at birth?

- Female (1)
- Male (3)

Q2 What is your ethnicity?

- White (1)
- Black or African American (2)
- American Indian or Alaska Native (3)
- Asian (4)
- Native Hawaiian or Pacific Islander (5)
- Hispanic / Latino (6)
- Other (7)

Q3 Are you a first-generation living in the United States?

- Yes (1)
- No (2)

Q4 What is your highest level of education?

- Middle school or less (1)
- High school / GED (2)
- 1-2 years college (3)
- 4 years college (4)
- Graduate / Professional school (5)

Q5 What is your zip code?

________________________________________________________________

Q6 Do you have strong support from your family and/or friends?

- Yes (1)
- No (2)

Q7 Do you attend a yearly female health appointment?

- Yes: family doctor (1)
- Yes: gynecologist (2)
- Yes: nurse (3)
- No (4)

Q37 Do you have health insurance?

- Yes (1)
- No (2)

Q8 Have you or anyone you know been diagnosed with cervical cancer and/or cervical dysplasia?

- Yes, I have. (1)
- Yes, I know someone who has. (2)
- No (3)

Q9 Do you have a close blood relative such as a mother, grandmother, aunt, first degree cousin, with ovarian, breast, pancreas, skin, or colon/rectal cancer?

- Yes (1)
- No (2)

Q10 Do you know that having a family member with cancer suggests a genetic risk for cancers in the family? Check all that apply.

- Yes, my doctor/nurse told me about it. (1)
- Yes, I read about it on social media. (2)
- Yes, I read about it in a magazine. (3)
- Yes, I heard about it on the radio. (4)
- Yes, my family / friend told me about it. (5)
- No (6)

Q11 If you answered "Yes" to Q10, have you ever gotten a genetic test to check your risk for cancer?

- Yes, my doctor/nurse ordered it for me. (1)
- Yes, I paid for an over-the-counter test such as 23&Me® (2)
- No, I do not care to know. (3)
- No, but I would like to learn more about it. (4)

Q12 Which one of the following would be easy or difficult for you to administer yourself at home?

|  | Easy (1) | Difficult (2) |
| --- | --- | --- |
| Insert a Q-tip-like swab into the vagina to collect a sample. (1) |  |  |
| Insert a Q-tip-like swab into the mouth to collect a sample. (2) |  |  |
| Send a FedEx or UPS Box (3) |  |  |
| Do an at-home flu, COVID-19 or RSV test from the nose. (4) |  |  |

Q13 Do you have and/or had any of the following cancers?

- Yes, I have/had ovarian cancer. (1)
- Yes, I have/had breast cancer. (2)
- Yes, I have/had uterine cancer. (3)
- Yes, I have/had cervical cancer. (4)
- No (5)

Q14 If you answered "Yes" to Q13, what types of testing and/or treatment did you receive? Select all that apply.

- Biomarker testing (1)
- Chemotherapy (2)
- Hormone therapy (3)
- Immunotherapy (4)
- Radiation therapy (5)
- Surgery (6)
- Targeted therapy (7)
- Clinical trial (8)

Q15 Have you ever heard of the term, "clinical trial"?

- Yes (1)
- No (2)

Q16 If you answered "Yes" to Q15, where did you hear about clinical trials?

________________________________________________________________

Q17 When do you think is the best time to learn about clinical trials?

- Before the person gets sick. (1)
- Only after the person gets sick. (2)

Q18 Who do you think is the most appropriate person to educate people about clinical trials?

- High school teacher (1)
- College professor (2)
- Primary care doctor / obstetrician / family medicine doctor (3)
- Nurse or medical personnel at the primary care provider's office (4)
- Specialist, such as a cardiologist, cancer oncologist (5)
- Local pharmacist (6)
- Other (7) __________________________________________________

Q19 Please answer "Agree" or "Disagree" to each question below, if you said you know what a clinical trial is.

|  | Agree (1) | Disagree (2) |
| --- | --- | --- |
| I am afraid of participating in a clinical trial. (1) |  |  |
| I would want my loved one with cancer to participate in a clinical trial. (2) |  |  |
| I know how to find a clinical trial for myself or for my loved one. (3) |  |  |
| I do not trust the health system / doctors / pharmaceuticals. (4) |  |  |
| I do not have time to participate in a clinical trial. (5) |  |  |
| I do not want to take medicines that have not been tested and/or proven. (6) |  |  |
| Participating in a clinical trial means I must spend money for medicines not covered by my insurance. (7) |  |  |
| Participating in a clinical trial means I must travel to a hospital and/or clinic far from my home. (8) |  |  |
| I do not want to have a new doctor whom I do not know. (9) |  |  |
| I do not want to fight against God's will for me. (10) |  |  |
| I do not have a reason to prolong my life. (11) |  |  |
| I will suffer more if I participate in a clinical trial. (12) |  |  |

Q20 Most people who participate in clinical trials are White. Why do you think White people participate in clinical trials, but African American / Black and Hispanic people do not?

________________________________________________________________

________________________________________________________________

________________________________________________________________

________________________________________________________________

________________________________________________________________

Q21 Who do you go to for advice on sexual and/or genital organ health?

- Family member (1)
- Obstetrician (2)
- Physician (3)
- Nurse (4)
- Online medical educator (5)
- Friend (6)
- Social media and/or internet posts (7)

Q22 If you answered, "Obstetrician", "Physician", or "Nurse" to Q21, would you feel equally as comfortable asking a male obstetrician, nurse, or physician about genital health advice, as a female one?

- Yes (1)
- No, I would feel more comfortable with a male. (2)
- No, I would feel more comfortable with a female. (3)

Q23 How much do you know about cancer and the way it works?

- Very knowledgeable (1)
- Knowledgeable (2)
- Somewhat knowledgeable (3)
- Not knowledgeable (4)

Q24 In your own opinion, how can providers improve the way they educate their patients?

________________________________________________________________

________________________________________________________________

________________________________________________________________

________________________________________________________________

________________________________________________________________

Q25 Is one annual visit with a provider sufficient or would you prefer additional educational visit(s) without an additional cost to you?

- Yes, one annual visit is sufficient. (1)
- No, it is difficult to get an appointment with my provider. I prefer a more convenient and faster appointment with a Health Educator. (2)
- No, I prefer to wait and have an additional appointment with my provider. (3)

Q26 About how much of the information provided to you by your health provider do you understand and remember after the visit?

- All (1)
- Most (2)
- Some (3)
- None (4)

Q28 Do you know:

|  | Yes (1) | No (2) |
| --- | --- | --- |
| Anyone close to you who participated in a clinical trial? (1) |  |  |
| How to find a clinical trial and join it. (2) |  |  |
| If you can leave a clinical trial at any time? (3) |  |  |

Q29 Would it be useful to you to hear from patients who have participated in clinical trials?

- Yes (1)
- No (2)
- I do not know (3)

Q30 How would you encourage someone with cancer to participate in a clinical trial? What would you tell them?

________________________________________________________________

________________________________________________________________

________________________________________________________________

________________________________________________________________

________________________________________________________________

End of Block: Pre-Education Questions

Start of Block: Please pause and shift your attention to your MiOra Health Ambassador.

Q36 Please pause and shift your attention to your MiOra Health Ambassador.

End of Block: Please pause and shift your attention to your MiOra Health Ambassador.

Start of Block: After Education

Q31 Do you feel like you know more about risk factors for women's cancers?

- Yes (1)
- No (2)

Q32 Do you feel like you know more about clinical trials, and you provided your input in the clinical trial diversity challenges?

- Yes (1)
- No (2)

Q33 Do you feel more comfortable participating in a clinical trial or recommending clinical trials to your friends and/or family?

- Yes (1)
- No (2)

Q34 If you'd like to get additional information on women's cancers and clinical trial, please enter your phone number, email, and/or social media below. This information is confidential and will not be shared.

- Phone number (1) __________________________________________________
- Email (2) __________________________________________________
- Social media handle (3) __________________________________________________

End of Block: After Education
